# Supplementary material for: Structural basis for SHOC2 modulation of RAS signalling
Source: Nature. 2022 Jun 29;609(7926):400–7. doi: 10.1038/s41586-022-04838-3 (PMC9452301; doi:10.1038/s41586-022-04838-3)
Supplement: Supplementary file 1 — List of cancer cell lines highly co-dependent on SHOC2 and HRAS, KRAS or NRAS. [file 41586_2022_4838_MOESM1_ESM.pdf]

nature portfolio

https://doi.org/10.1038/s41586-022-04838-3

Supplementary information

Structural basis for SHOC2 modulation of RAS signalling

In the format provided by the authors and unedited

Supplementary Information Table 1: List of cell lines highly co-dependent on SHOC2 and KRAS, NRAS or HRAS according to DepMap CRISPR KO dataset. Cell lines are defined as highly codependent when Chronos scores are both less than -0.75 (indicated in red).

N	Cell line name	KRAS chronos	NRAS chronos	HRAS chronos	SHOC2 chronos	Lineage	Hugo Symbol	Protein Change
1	IPC298	-0.14	**-1.30**	-0.11	**-1.29**	skin	NRAS	p.Q61L
2	NB4	**-1.53**	-0.15	-0.11	**-1.17**	blood	KRAS	p.A18D
3	AML193	-0.33	**-1.75**	-0.15	**-1.17**	blood	NRAS	p.G13V
4	RPE1SS6	**-1.62**	-0.13	-0.16	**-1.17**	engineered		
5	SHI1	**-1.17**	0.035	-0.21	**-1.13**	blood	KRAS	p.Q61H
6	G415	**-1.56**	0.23	0.11	**-1.11**	bile_duct	KRAS	p.G13D
7	LS1034	**-1.76**	-0.31	-0.08	**-1.08**	colorectal	KRAS	p.A146T
8	SKMEL2	-0.31	**-1.33**	-0.07	**-1.08**	skin	NRAS	p.Q61R
9	HT1080	-0.31	**-1.42**	-0.20	**-1.04**	soft_tissue	NRAS	p.Q61K
10	SNU620	**-1.46**	-0.16	-0.27	**-1.04**	gastric		
11	CHP212	-0.48	**-1.13**	-0.03	**-1.01**	peripheral_nervous_system	NRAS	p.Q61K
12	CALU6	**-1.61**	-0.08	-0.04	**-1.0**	lung	KRAS	p.G60G
12	CALU6	**-1.61**	-0.08	-0.04	**-1.0**	lung	KRAS	p.Q61K
13	ICC3	**-1.04**	-0.22	-0.34	**-0.99**	bile_duct	KRAS	p.G12D
14	ONS76	-0.25	**-0.96**	-0.07	**-0.99**	central_nervous_system	NRAS	p.Q61R
15	NCIH1915	-0.32	-0.27	**-1.05**	**-0.98**	lung	HRAS	p.Q61L
16	CCLFUPGI0005T	**-1.24**	-0.21	-0.16	**-0.97**	gastric		
17	TT2609C02	-0.29	**-1.57**	-0.06	**-0.96**	thyroid	NRAS	p.Q61R
18	BE2M17	**-0.76**	-0.06	-0.06	**-0.94**	peripheral_nervous_system		
19	HS936T	-0.48	**-1.59**	-0.24	**-0.93**	skin	NRAS	p.Q61K
20	NCIH2087	-0.38	**-1.13**	-0.12	**-0.91**	lung	NRAS	p.Q61K
21	NCIH2087	-0.38	**-1.13**	-0.12	**-0.91**	lung	NRAS	p.A59A
22	RCM1	**-1.86**	0.11	-0.03	**-0.91**	colorectal	KRAS	p.G12V

23	HEPG2	-0.43	**-1.72**	-0.09	**-0.90**	liver	NRAS	p.Q61L
24	MM127	-0.40	**-1.58**	-0.32	**-0.88**	skin		
25	SKMEL30	-0.19	**-1.44**	-0.20	**-0.87**	skin	NRAS	p.G60G
25	SKMEL30	-0.19	**-1.44**	-0.20	**-0.87**	skin	NRAS	p.Q61K
26	HS766T	**-1.09**	-0.24	-0.15	**-0.85**	pancreas	KRAS	p.Q61H
27	MM485	-0.45	**-1.04**	-0.38	**-0.83**	skin	NRAS	p.Q61R
28	TYKNU	-0.08	**-1.37**	-0.24	**-0.83**	ovary	NRAS	p.Q61K
28	TYKNU	-0.08	**-1.37**	-0.24	**-0.83**	ovary	NRAS	p.G12D
29	LI7	**-0.82**	-0.29	-0.09	**-0.81**	liver		
30	DIFI	**-1.32**	-0.29	0.00	**-0.79**	colorectal		
31	MELJUSO	-0.41	**-1.45**	-0.88	**-0.79**	skin	HRAS	p.G13D
31	MELJUSO	-0.41	**-1.45**	-0.88	**-0.79**	skin	NRAS	p.Q61L
32	RBE	**-1.06**	0.00	-0.16	**-0.79**	bile_duct	KRAS	p.G12V
33	RPE1SS48	**-1.38**	-0.36	-0.46	**-0.76**	engineered		
34	SW579	**-1.43**	-0.06	0.10	**-0.76**	thyroid		
