# Supplementary material for: Structural basis for SHOC2 modulation of RAS signalling
Source: Nature. 2022 Jun 29;609(7926):400–7. doi: 10.1038/s41586-022-04838-3 (PMC9452301; doi:10.1038/s41586-022-04838-3)
Supplement: Supplementary file 2 — Reporting Summary [file 41586_2022_4838_MOESM2_ESM.pdf]

nature portfolio

Corresponding author(s): Sarah G. Hymowitz, Jawahar Sudhamsu

Last updated by author(s): Apr 13, 2022

Reporting Summary

Nature Portfolio wishes to improve the reproducibility of the work that we publish. This form provides structure for consistency and transparency in reporting. For further information on Nature Portfolio policies, see our Editorial Policies and the Editorial Policy Checklist.

Statistics

For all statistical analyses, confirm that the following items are present in the figure legend, table legend, main text, or Methods section.

n/a	Confirmed	
☐	☒	The exact sample size (*n*) for each experimental group/condition, given as a discrete number and unit of measurement
☐	☒	A statement on whether measurements were taken from distinct samples or whether the same sample was measured repeatedly
☐	☒	The statistical test(s) used AND whether they are one- or two-sided<br>*Only common tests should be described solely by name; describe more complex techniques in the Methods section.*
☒	☐	A description of all covariates tested
☒	☐	A description of any assumptions or corrections, such as tests of normality and adjustment for multiple comparisons
☐	☒	A full description of the statistical parameters including central tendency (e.g. means) or other basic estimates (e.g. regression coefficient) AND variation (e.g. standard deviation) or associated estimates of uncertainty (e.g. confidence intervals)
☐	☒	For null hypothesis testing, the test statistic (e.g. *F*, *t*, *r*) with confidence intervals, effect sizes, degrees of freedom and *P* value noted<br>*Give P values as exact values whenever suitable.*
☒	☐	For Bayesian analysis, information on the choice of priors and Markov chain Monte Carlo settings
☒	☐	For hierarchical and complex designs, identification of the appropriate level for tests and full reporting of outcomes
☐	☒	Estimates of effect sizes (e.g. Cohen's *d*, Pearson's *r*), indicating how they were calculated

Our web collection on statistics for biologists contains articles on many of the points above.

Software and code

Policy information about availability of computer code

Data collection	SerialEM 3.7.11.
Data analysis	Prism8 (GraphPad); UCSF ChimeraX 1.2.5; Phenix 1.18; The ConSurf Server (https://consurf.tau.ac.il/); PyMOL 2.3.2 (The PyMOL Molecular Graphics System, Schrödinger, LLC); Coot 0.89; UniDec 4.4.0; CryoSPARC 3.0.1; CryoSPARC Live 3.1; MODELLER 9.21; BILBOMD 1.0; FoXS 2.1; FF19SB; Gaussian09; Amber2018; CPPTRAJ 18.0.

For manuscripts utilizing custom algorithms or software that are central to the research but not yet described in published literature, software must be made available to editors and reviewers. We strongly encourage code deposition in a community repository (e.g. GitHub). See the Nature Portfolio guidelines for submitting code & software for further information.

Data

Policy information about availability of data

All manuscripts must include a data availability statement. This statement should provide the following information, where applicable:

- Accession codes, unique identifiers, or web links for publicly available datasets
- A description of any restrictions on data availability
- For clinical datasets or third party data, please ensure that the statement adheres to our policy

Coordinates and related data for SHOC2 and SHOC2:PP1C:MRAS complex have been deposited in the PDB and EMDB respectively: PDB 7DS1 for SHOC2 and PDB 7DS0 and EMDB-25044 for the SHOC2:PP1C:MRAS complex. SAXS data and atomistic models have been deposited at the SASBDB database as entries SDSDMB5 (SHOC2), SDSDMC5 (SHOC2:PP1C), SDSDMD5 (SHOC2:PP1C:KRAS) and SASDME5 (SHOC2:PP1C:MRAS).

DepMap release Public 21Q4 datasets containing cell line information (sample_info.csv), chronos scores (CRISPR_gene_effect.csv) and mutational status

March 2021

(CCLE_mutations.csv), are publicly available from https://depmap.org/portal/download/.

AlphaFold Protein Structure Database (https://alphafold.ebi.ac.uk)

Other data that support the findings of this study are available from the corresponding author upon request.

Field-specific reporting

Please select the one below that is the best fit for your research. If you are not sure, read the appropriate sections before making your selection.

☒ Life sciences ☐ Behavioural & social sciences ☐ Ecological, evolutionary & environmental sciences

For a reference copy of the document with all sections, see nature.com/documents/nr-reporting-summary-flat.pdf

Life sciences study design

All studies must disclose on these points even when the disclosure is negative.

Sample size	For enzyme assays, n=2 was chosen for technical replicates to ensure linearity of reactions over time. For binding assays, n=2 was chosen for technical replicates to ensure binding equilibrium had been reached. For X-ray structure determination, only one diffracting crystal was ever obtained for analysis. For CryoEM data collection, sample size was not pre-determined. The number of micrographs collected was determined by microscope availability and sufficient resolution being reached in the final structure to make functional conclusions.
Data exclusions	All attempts at replication were successful. No replicates were excluded from analysis for any experiments.
Replication	Biochemical assays were performed as three independent experiments. Within each independent experiment two time points were samples (technical replicates).
Randomization	For the X-ray structure, a random Rfree set of data was excluded for later validation of the model. For the CryoEM structure, two random half data sets were independently refined to provide resolution cutoff estimates.
Blinding	Blinding is not applicable to this study. Blinding was not performed in this study because it is not necessary or practical for X-ray or CryoEM structural determination.

Reporting for specific materials, systems and methods

We require information from authors about some types of materials, experimental systems and methods used in many studies. Here, indicate whether each material, system or method listed is relevant to your study. If you are not sure if a list item applies to your research, read the appropriate section before selecting a response.

Materials & experimental systems

n/a	Involved in the study	
☒	☐	Antibodies
☐	☒	Eukaryotic cell lines
☒	☐	Palaeontology and archaeology
☒	☐	Animals and other organisms
☒	☐	Human research participants
☒	☐	Clinical data
☒	☐	Dual use research of concern

Methods

n/a	Involved in the study	
☒	☐	ChIP-seq
☒	☐	Flow cytometry
☒	☐	MRI-based neuroimaging

Eukaryotic cell lines

Policy information about cell lines

Cell line source(s)	Sf9 cell line (insect cells) was used for over-expression of some of the proteins used in this study which were then purified from the cell using chromatography techniques. Sf9 cells were obtained from Expression Systems (https://expressionsystems.com/)
Authentication	The cell line was not authenticated independently.
Mycoplasma contamination	The cell line was not tested for mycoplasma contamination.
Commonly misidentified lines (See ICLAC register)	Not Applicable
